# Image-Based Communication for Strengthening Patient Health Education in Rural and Underserved Settings

**DOI:** 10.7759/cureus.41279

**Published:** 2023-07-02

**Authors:** Rakesh R Tiwari, Bhrigupati Pandey, Kaustubh S Chaudhari

**Affiliations:** 1 Department of Ayurved Basic Principles (Department of Ayurveda Samhita Siddhanta), Dr. D.Y. Patil College of Ayurved & Research Centre, Dr. D.Y. Patil Vidyapeeth (Deemed to be University), Pune, IND; 2 Department of Ayurved Basic Principles (Department of Ayurveda Samhita Siddhanta), K.G. Mittal Ayurved College, Mumbai, IND; 3 Department of Internal Medicine, Dr. Vaishampayan Memorial Government Medical College, Solapur, IND

**Keywords:** image-based communication, pictures, health communication, patient education, public health

## Abstract

Effective communication is the cornerstone of efficient patient care. It is vital to obtain a thorough history, build the patient’s trust, and ensure compliance to treatment. Image-based communication (IBC) using comic-like strips is better than the conventional verbal and written modes, as it is inexpensive, less human resource dependent, and diversity agnostic. Strips based on local and socioculturally relevant issues and characters grab readers’ attention, are relatable and entertaining, and utilize a storyline that invigorates thinking. The medical advice delivered by an ideal IBC strip is easy to comprehend, has a better recall, and promotes patient adherence. With an idea that IBC strips can serve as a vital supportive tool in underserved and overburdened clinics, we have described the nuances of adapting them into the existing physician-patient experience. We utilize a prototype IBC of an elderly woman helping a family whose child developed acute fever, possibly malaria. Various elements of an IBC strip, namely, panels, gutters, background, characters, bubbles, captions, and visual effects, are illustrated, and their variations are described later. Once designed, an IBC strip must be critically evaluated for the accuracy of the educational message, and errors, if any, must be corrected. The images are then subjected to a series of local field tests to ensure that they serve their purpose and have the desired cultural competence. Once ready, IBC strips can be posted in public spaces and outside clinics or distributed to healthcare workers or patients. Here, they serve as educational and health literacy tools. The strips can significantly reduce caregiver-patient interaction time and improve the quality of communication, especially when patients are illiterate or understand a different language. It is easier to develop rapport and partnership with a patient when the communication is presented through a pictorial tool. An IBC strip can be used to train grassroot workers, who subsequently train patients, thereby serving a dual purpose. To obtain tangible clinical and epidemiologic benefits from IBC strips, rigorous evidence building and standardization are a crucial long-term goal.

## Introduction

Medical practice runs on the premise of informed decision making where information is provided by a physician and the patient or guardian takes a decision accordingly. Hence, effective communication is the cornerstone of efficient patient care. The constrained doctor-patient ratio, an increased need to seek clinical evidence, and the cumbersome paperwork have markedly reduced the time doctors get to spend with their patients face to face. This reduces the opportunity to counsel and explain patients about their condition [1]. Inequality exists in treatments based around verbal communication [2] because of the geographical, educational, linguistic, and sociocultural divide between the physician and their patients [3]. In low-resource rural and urban settings, patients are often cared for by physicians, nurses, and paramedical professionals from other locations. This further reduces the fluency of communication with patients. While speaking, physicians often use medical jargon that they learnt in medical school to communicate with their patients. Such explanations often supersede patients’ level of understanding, especially more so with illiterate patients [4].

Effective communication is essential to gain a thorough patient history, keep patients informed, build their trust, and ensure compliance to the suggested treatment. A conventional doctor-patient interaction takes place in a clinic or a home-based setting where communication is either written or verbal [4]. This however is constrained by the aforementioned reasons.

Other modes of communication have their own constraints. For example, counseling patients through nurses, paramedical professionals, call center channels, and grassroot workers is human resource intensive and hence at times expensive [5,6]. Communication using e-mails, text messages, phone calls, and brochures depends on a certain level of literacy and availability of electronic devices, which can be expensive and difficult to use. Infotainment videos, although very effective, are difficult to produce, might require professional voiceover, and require a longer production time [7]. These indirect modes of communication lack the human touch vital for clinical practice.

Augmented communication blended with clinical practice not only reduces doctor-patient interaction durations but also improves the quality of the consultation and consequently the patient satisfaction rates. Image-based communication (IBC) strips have an immense potential for becoming a supporting communication tool in underserved and overburdened clinics. We have described the nuances of adapting an IBC technique integrated with the conventional clinical experience to address some of the concerns raised above.

## Technical report

The current medical practice does involve use of images in communication; however, their use is limited, sporadic, and not standardized. The commonly used image types are photographs, posters, sketches, doodles, comic strips, mimes, and sand paintings.

The use of photographs makes pictures more realistic and easier to imagine. Cancer images have been successfully used to promote tobacco and smoking de-addiction [8]. However, it can be cumbersome and expensive to create real-life healthcare scenarios for photographing. Sketches are easy to draw and can be created on the field too. They can be used to simplify difficult concepts and make clinical discussions more engaging. Solitary sketches however cannot deliver a storyline. Sand paintings are considered auspicious in many communities. One such tradition, the rangoli, has been used to promote healthcare advocacy and learn about local issues through local competitions [9]. Comics, doodles, and memetics consist of catchy images that utilize humor to deliver vital healthcare advice.

IBC strips are like comics. They use a storyline, but unlike comics, they are not always funny. IBC strips often integrate the advantages and bridge the gaps of the other modalities. They are entertaining, utilize relatable characters, can deliver a storyline, invigorate a thinking process, and are a great tool for passive and/or integrative learning. An effective IBC strip must illustrate healthcare issues and advice that is relevant to the population it targets. Before understanding how to adapt an image to deliver healthcare advice, let us understand the basic elements of a good IBC strip.

In an IBC strip, images are presented in the form of "panels." Each panel describes an event that is in continuum with the panels preceding and succeeding it. When the panels are arranged in a sequence, a "storyline" is created. Each panel is separated from adjacent panels by means of a narrow blank space called the "gutter." A panel has "characters," which are the people (real or fictitious) who are a part of the storyline. They are presented against a "background" that indicates where the story takes place. A good background must not be cluttered to avoid distraction. "Bubbles" and "captions" have text that indicate what the characters are speaking. "Sound effects" are onomatopoeia words that can be included in images to indicate motion. For example, the sound "swoosh" can be written behind a car to indicate that it is moving fast. "Visual effects" are symbols or strokes that can be used to indicate actions. For example, the characters "zzz" can be written opposite a person lying down to indicate that they are asleep.

**Figure 1:**
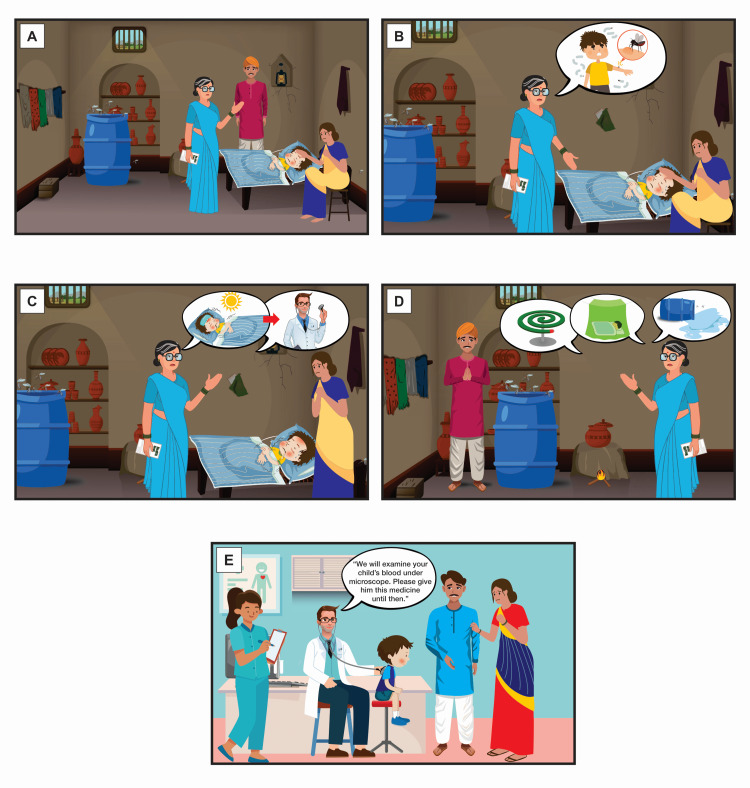
Image-based communication (IBC) strip illustrating a malaria patient receiving local and medical advice The characters, individual components, and storyline are described in detail in the article.

As an example (Figure 1), we describe an IBC strip conceptualized and designed by the lead investigator of the study (author initials: KSC). The IBC shows an elderly woman helping a family whose child developed acute fever with chills. They receive local and professional medical advice for a possible malaria infection in the five panels of the storyline. The scenarios are presented in an imaginary hybrid rural-urban setup to help readers compare images made for the respective demographics. The first panel (Figure 1A) depicts the problem. It shows a respected, elderly woman, like a healthcare worker (HCW) or school teacher in the village visiting a rural household to attend to a child with fever. The parents are depicted to look worried, but they pay keen attention to the HCW’s instructions. The mother is shown sitting beside the sleeping child. The rural setup is indicated by a mud walled house and a traditional cot on which the child is sleeping. The child is covered by a warm blanket, but he continues to shiver as indicated by the vibration visual effect around him. The face is shaded with a reddish hue to indicate fever. The father is standing in the background. In the second panel (Figure 1B), there is a speech bubble with an image of a mosquito biting the child, to indicate the HCW explaining to the mother why her child may have developed fever.

The third panel (Figure 1C) shows home-based care suggestions that are a routine practice in rural setup. The mother is seen measuring the child's fever with a thermometer while receiving instructions on cold sponging from the HCW. The HCW recommends visiting a doctor if the fever does not resolve by morning. This is depicted by speech bubbles with images of the sun, a feverish and drowsy child, and an arrow pointing towards a doctor. The fourth panel (Figure 1D) indicates other preventive measures suggested by the HCW. She advises the father to empty the can full of water as it can harbor mosquitoes. She also asks him to procure mosquito coils and mosquito net, both depicted in a speech bubble.

A shift of environment from rural to urban is made for the next panel (Figure 1E) to provide a comparison as indicated above. The child with unremitting fever is now taken to a clinic in a city where the doctor, after examination, recommends a microscopic blood examination and some medicines in "text bubble" vis-a-vis an "image bubble" used before. The panel numbering is in accordance with journal image referencing requirements. In a real-life scenario, because illiterate persons cannot read the numbers, the image panels are arranged in sequence or connected by arrows.

Once any IBC strip is contextualized as explained in the above example, it must be evaluated critically by first examining the image or panel as a whole followed by quadrant-wise and element-wise inspection. All characters and elements must be vital to the image and continuum. Unnecessary or redundant characters must be removed from the panels. The bubbles or captions must be concise and target-specific.

Once finalized, the panels are printed in size large enough to keep all the elements legible. These prints are then subjected to a field test, first with educated and elderly people in the target population followed by a subset of the population. Their critical inputs must then be incorporated into the IBC strip before it is sent for final printing. This method of the subject "teaching the teacher" is crucial for sociocultural tailoring of the image for its target audience. The nuances introduced into the originally approved strip are specific to the specific patient groups and must be adopted for different groups.

Once ready, the images can be placed in residential areas, schools, public spaces, and outside clinics. They can also be distributed among doctors and other HCWs as a supplementary tool for their consultation.

## Discussion

The main goal of using images, especially IBC strips, is to strengthen the message delivered in clinical advice, both preventive and therapeutic. The heterogeneity of medical issues and the sociocultural variations of people dealing with them makes medical communication increasingly difficult [4]. There has been significant progress in communicating with patients with disability [10], but discussing medical advice with illiterate and innumerate populations still remains a challenge [11]. Through this report, we intend to guide the development of image-based tools to address this concern.

A good clinical image must ensure attention, comprehension, recall, and adherence by a patient [4]. As in our example, most major recognition signs, preventive measures, and diagnostic and therapeutic decisions must be conveyed to the patient through an IBC to make it "comprehensive." An IBC strip can include known characters and common scenarios to grab the patient's "attention." The storyline depicted in the IBC strip automatically ensures a good "recall." Patient "adherence" is more likely, when the characters and stories presented in the image are relatable and the message is comprehensive [4].

Just as the utilization of known characters is vital for a good image, it helps if their advice is in a speech bubble and not a caption [4]. Using colored images is preferable if feasible. Flow charts that indicate continuity help faster delivery of messages. The use of numbers and text makes IBC very meaningful, but they must be replaced by symbols for illiterate and innumerate populations. Symbols are also useful for communicating with people speaking different languages. For example, the "sun" is used to depict "morning" for caregivers who could not check the time on a clock (innumerate), and the mosquito coil image is used for those who cannot read (illiterate).

In underserved settings, there is a lot of reliance on grassroot workers for healthcare delivery, which can be challenging. For example, traditional birth attendants (TBAs) who visit houses for home deliveries can hardly find help in case of complicated pregnancies. Hence, it is vital to train the TBAs about the danger signs of pregnancy, such as abdominal signs, vaginal bleeding, fever, and convulsions. As many of these TBAs lack formal education, using images to explain about these signs can be useful.

Previously, TBAs and auxiliary nurse midwives (ANMs) used photographs and dolls to train patients for their delivery and neonatal care [12]. Similarly, neonatal nurses were trained in kangaroo mother care using mannequins [13]. IBC strips can serve as an inexpensive alternative to training healthcare workers, who can subsequently use the same strips to train expectant mothers and their caregivers.

Posters have been extensively used to spread awareness about maternal and child health issues, infectious diseases, and healthy practices [14]. Integrating the messages from posters in a series of IBC strips can make communication fluent and explanations simpler. Using image strips can also simplify clinical consultations by using the principles of the RESPECT model (i.e., Rapport, Empathy, Support, Partnership, Explanations, Cultural competence, and Trust) [15]. An image with culturally relatable characters is an effective explanation tool. While images make explanations easier, relatable images help establish both trust and rapport. Explaining health issues through third-party cartoons becomes easier for the patients too, thereby ensuring partnership. Patients can be given takeaway IBC strips, which can provide in-home support and guidance.

IBC strips are simple to design, modifiable at the source, and relatively inexpensive and can be easily posted and circulated. However, unlike animation and movies, they lack expressivity and are less natural. An important challenge while using IBC strips is to ensure standardization while repetitively customizing them. Validating the images by performing field tests and tallying them with a clinician are crucial to ensure uniformity of the message being conveyed.

## Conclusions

IBC strips, despite being still images, can be a very effective communication tool in clinical practice, both at the bedside and in the field. When used in conjunction with conventional consultations, they help establish rapport, provide simple explanations, suggest lifestyle modifications, and explain treatment options to the patients with ease. They are inexpensive, can be socioculturally tailored, and deliver a storyline easily understood by a layperson. This makes them a very effective communication tool, especially in an underserved healthcare setting. Extensive field research on the tangible public health benefits of IBC strips is crucial for their success.
